# Supplementary material for: Importance of attributes and willingness to pay for oral anticoagulant therapy in patients with atrial fibrillation in China: A discrete choice experiment
Source: PLoS Med. 2021 Aug 26;18(8):e1003730. doi: 10.1371/journal.pmed.1003730 (PMC8432810; doi:10.1371/journal.pmed.1003730)
Supplement: S1 File — (DOCX) [file pmed.1003730.s001.docx]

**S1 File. Sample for scenario 1 (for the purpose of testing patients’ understanding)**

Imagine you are choosing an oral anticoagulant therapy for stroke prevention. Please choose your favorite **ONE** from “Drug A”, “Drug B” and “Do not accept any oral anticoagulant therapy” according to the profiles of provided alternatives in each scenario. **Your choice will not affect your current or later treatment.**

| **Scenario 1** | **Drug A** | **Drug B** | **Do not accept any oral anticoagulant therapy** |
| --- | --- | --- | --- |
| Antidote | No | Yes | Not available |
| Food-drug interaction | Yes | No | Not available |
| Frequency of blood monitoring | Every 3 months | No need | Not available |
| Risk of major bleeding | 7.8% | 3.1% | Background risk |
| Risk of stroke (ischemic/hemorrhage) or systemic embolism | 8.4% | 5.8% | Background risk |
| Risk of acute myocardial infarction | 1.0% | 1.0% | Background risk |
| Out-of-pocket (RMB/month) | 240 | 0 | 0 |
| Which one would you prefer |  |  |  |

RMB indicates Ren Min Bi, the unit of Chinese currency.
