# Supplementary material for: Importance of attributes and willingness to pay for oral anticoagulant therapy in patients with atrial fibrillation in China: A discrete choice experiment
Source: PLoS Med. 2021 Aug 26;18(8):e1003730. doi: 10.1371/journal.pmed.1003730 (PMC8432810; doi:10.1371/journal.pmed.1003730)
Supplement: S2 File — (DOCX) [file pmed.1003730.s002.docx]

**S2 File. Summary of the D-Efficient design using Ngene**

A D-Efficient design can yield data that enables estimation of the parameters (patients’ preference) with lowest possible standard errors, which can be predicted by determining the asymptotic variance-covariance (AVC) matrix based on the underlying experiment and some prior information about the parameter estimates (1). Prior parameters obtained from Ghijben et al’s study (2) are shown below.

| **Attribute** | **Coefficient (SE)** |
| --- | --- |
| Antidote | 0.637 (0.115) |
| Food-drug interaction | -0.404 (0.099) |
| Frequency of blood monitoring | -0.04 (0.078) |
| Risk of bleeding | 0.0636 (0.072) |
| Risk of stroke | 0.662 (0.073) |
| Risk of AMI* | 0.6 (NA) |
| Out-of-pocket monthly cost | -0.015 (0.002) |

SE indicates standard error; AMI indicates acute myocardial infarction.

*AMI was not included in Ghijben et al’s study; 0.6, which approximates the risk of bleeding and stroke, as the prior parameter for AMI in efficient design.

Multinomial logit model was used to evaluate the efficiency measures. The optimization for the variance of the ratio of two parameters (for mWTP calculation) was also considered. Most of the prior parameters were treated as normal distributed random effects except for risk of AMI, for which only the fixed effect was considered.

**Ngene coding for D-Efficient design:**

Design

;alts = DrugA*, DrugB*, DrugC

;rows = 16

;block = 2

;eff = (mnl, wtp(ref1))

;wtp = ref1(*/b8)

;model:

U(DrugA) = b1 +

b2[(n,0.637,0.115)] * antidote[0,1] +

b3[(n,-0.404,0.099)] * food[0,1] +

b4[(n,-0.04,0.078)] * test[0,1,2,3] +

b5[(n,0.636,0.072)] * bleed[0,1,2,3] +

b6[(n,0.662,0.073)] * stroke[0,1,2,3] +

b7[0.6] * ami[0,1,2,3] +

b8[(n,-0.015,0.002)] * price[0,1,2,3] /

U(DrugB) = b2 * antidote+

b3 * food +

b4 * test +

b5 * bleed +

b6 * stroke +

b7 * ami +

b8 * price$

Reference

1. ChoiceMetrics L. Ngene 1.1.2 USER MANUAL & REFERENCE GUIDE. 2014.

2. Ghijben P, Lancsar E, Zavarsek S. Preferences for oral anticoagulants in atrial fibrillation: a best-best discrete choice experiment. Pharmacoeconomics. 2014;32(11):1115-27.
