# Supplementary material for: Importance of attributes and willingness to pay for oral anticoagulant therapy in patients with atrial fibrillation in China: A discrete choice experiment
Source: PLoS Med. 2021 Aug 26;18(8):e1003730. doi: 10.1371/journal.pmed.1003730 (PMC8432810; doi:10.1371/journal.pmed.1003730)
Supplement: S3 File — (DOCX) [file pmed.1003730.s003.docx]

**S3 File. Results of balance for alternatives in designed questionnaire.**

| **Block** | **Scenario** | **Drug** | **Antidote** | **Food-drug** | **Monitoring** | **Bleeding** | **Stroke** | **AMI** | **Cost** | **Dominant checking*** |
| --- | --- | --- | --- | --- | --- | --- | --- | --- | --- | --- |
| 1 | 1 | A | 1 | 1 | 3 | 3 | 2 | 0 | 0 | 3 |
|  |  | B | 1 | 0 | 0 | 0 | 3 | 1 | 3 | 3 |
|  | 2 | A | 0 | 1 | 2 | 0 | 0 | 0 | 1 | 1 |
|  |  | B | 0 | 1 | 1 | 1 | 0 | 0 | 2 | 2 |
|  | 3 | A | 0 | 0 | 1 | 1 | 1 | 2 | 2 | 1 |
|  |  | B | 1 | 0 | 2 | 1 | 2 | 2 | 1 | 3 |
|  | 4 | A | 0 | 0 | 3 | 2 | 3 | 3 | 3 | 3 |
|  |  | B | 0 | 0 | 1 | 3 | 3 | 2 | 0 | 1 |
|  | 5 | A | 1 | 0 | 1 | 3 | 2 | 2 | 3 | 3 |
|  |  | B | 1 | 0 | 0 | 2 | 2 | 3 | 0 | 1 |
|  | 6 | A | 1 | 1 | 0 | 0 | 3 | 3 | 1 | 3 |
|  |  | B | 1 | 0 | 3 | 3 | 1 | 1 | 2 | 3 |
|  | 7 | A | 0 | 1 | 2 | 0 | 1 | 0 | 2 | 2 |
|  |  | B | 0 | 1 | 3 | 0 | 0 | 0 | 1 | 1 |
|  | 8 | A | 1 | 0 | 0 | 3 | 0 | 1 | 0 | 2 |
|  |  | B | 0 | 1 | 1 | 2 | 1 | 3 | 3 | 5 |
| 2 | 1 | A | 0 | 1 | 0 | 2 | 2 | 2 | 0 | 1 |
|  |  | B | 1 | 1 | 1 | 3 | 2 | 0 | 3 | 4 |
|  | 2 | A | 1 | 0 | 1 | 3 | 1 | 3 | 1 | 1 |
|  |  | B | 1 | 0 | 2 | 1 | 3 | 3 | 2 | 3 |
|  | 3 | A | 1 | 0 | 2 | 1 | 3 | 2 | 1 | 2 |
|  |  | B | 1 | 1 | 0 | 2 | 2 | 2 | 2 | 3 |
|  | 4 | A | 1 | 1 | 3 | 2 | 2 | 3 | 0 | 3 |
|  |  | B | 1 | 0 | 0 | 3 | 0 | 3 | 3 | 2 |
|  | 5 | A | 0 | 0 | 1 | 2 | 1 | 1 | 3 | 2 |
|  |  | B | 0 | 0 | 2 | 1 | 1 | 2 | 0 | 2 |
|  | 6 | A | 1 | 1 | 3 | 0 | 0 | 1 | 2 | 2 |
|  |  | B | 0 | 1 | 3 | 0 | 1 | 1 | 1 | 1 |
|  | 7 | A | 0 | 0 | 0 | 1 | 3 | 1 | 3 | 1 |
|  |  | B | 0 | 1 | 2 | 2 | 3 | 1 | 0 | 3 |
|  | 8 | A | 0 | 1 | 2 | 1 | 0 | 0 | 2 | 2 |
|  |  | B | 0 | 1 | 3 | 0 | 0 | 0 | 1 | 1 |

Food-drug indicates food-drug interaction; monitoring indicates the frequency of blood monitoring; bleeding indicates the risk of major bleeding; stroke indicates the risk of stroke (ischemic/hemorrhagic) or systemic embolism; AMI indicates the risk of acute myocardial infarction; cost indicates monthly out-of-pocket cost. RMB indicates Ren Min Bi, the unit of Chinese currency.

*The value in dominant checking represents the number of dominated attributes for one alternative in specific scenarios. All levels of the attributes would be the same if the values were all 0 in the same scenario (which is impossible for trade-offs). One alternative would be dominated if its value was greater than 0, and the other one was 0.

Levels for antidote and food-drug interaction is indicated by “Yes” if 1, “No” if 0; levels for the frequency of blood monitoring is indicated by “no need” if 0, “every 6 months” if 1, “every 3 months” if 2, “every 1 month” if 3; levels for the risk of major bleeding is indicated by “0.7%” if 0, “3.1%” if 1, “5.5%” if 2, “7.8%” if 3; levels for the risk of stroke or systemic embolism is indicated by “0.6%” if 0, “3.2%” if 1, “5.8%” if 2, “8.4%” if 3; levels for the risk of acute myocardial infarction is indicated by “0.2%” if 0, “1.0%” if 1, “1.8%” if 2, “2.5%” if 3; levels for out-of-pocket is indicated by “0 RMB” if 0, “120 RMB” if 1, “240 RMB” if 2, “360 RMB” if 3.
