# Supplementary material for: Importance of attributes and willingness to pay for oral anticoagulant therapy in patients with atrial fibrillation in China: A discrete choice experiment
Source: PLoS Med. 2021 Aug 26;18(8):e1003730. doi: 10.1371/journal.pmed.1003730 (PMC8432810; doi:10.1371/journal.pmed.1003730)
Supplement: S4 File — (DOCX) [file pmed.1003730.s004.docx]

**S4 File. Patients’ responses.**

| **BLK** |  | **Drug A** | | | | | | | **Drug B** | | | | | | | **None**  **N (%)** | **A**  **N (%)** | **B**  **N (%)** | **Total** |
| --- | --- | --- | --- | --- | --- | --- | --- | --- | --- | --- | --- | --- | --- | --- | --- | --- | --- | --- | --- |
|  | SCE | ATD | F-D | MONI | BLD | STK | AMI | COST | ATD | F-D | MONI | BLD | STK | AMI | COST |  |  |  |  |
| 1 | 1 | Yes | Yes | Per 1 month | 0.7% | 3.2% | 2.5% | 0 | Yes | No | No need | 7.8% | 0.6% | 1.8% | 360 | 16 (6.3) | 121 (47.6) | 113 (44.5) | 254 |
|  | 2 | No | Yes | Per 3 months | 7.8% | 8.4% | 2.5% | 120 | No | Yes | Per 6 months | 5.5% | 8.4% | 2.5% | 240 | 18 (7.1) | 38 (15.0) | 194 (76.4) | 254 |
|  | 3 | No | No | Per 6 months | 5.5% | 5.8% | 1.0% | 240 | Yes | No | Per 3 months | 5.5% | 3.2% | 1.0% | 120 | 10 (3.9) | 27 (10.6) | 213 (83.9) | 254 |
|  | 4 | No | No | Per 1 month | 3.1% | 0.6% | 0.2% | 360 | No | No | Per 6 months | 0.7% | 0.6% | 1.0% | 0 | 5 (2.0) | 39 (15.4) | 206 (81.1) | 254 |
|  | 5 | Yes | No | Per 6 months | 0.7% | 3.2% | 1.0% | 360 | Yes | No | No need | 3.1% | 3.2% | 0.2% | 0 | 7 (2.8) | 119 (46.9) | 124 (48.8) | 254 |
|  | 6 | Yes | Yes | No need | 7.8% | 0.6% | 0.2% | 120 | Yes | No | Per 1 month | 0.7% | 5.8% | 1.8% | 240 | 15 (5.9) | 165 (65.0) | 70 (27.6) | 254 |
|  | 7 | No | Yes | Per 3 months | 7.8% | 5.8% | 2.5% | 240 | No | Yes | Per 1 month | 7.8% | 8.4% | 2.5% | 120 | 29 (11.4) | 179 (70.5) | 42 (16.5) | 254 |
|  | 8 | Yes | No | No need | 0.7% | 8.4% | 1.8% | 0 | No | Yes | Per 6 months | 3.1% | 5.8% | 0.2% | 360 | 13 (5.1) | 99 (39.0) | 138 (54.3) | 254 |
|  | Total^*^ |  |  |  |  |  |  |  |  |  |  |  |  |  |  | 113 (5.6) | 787 (38.7) | 1100 (54.1) | 2032 |
| 2 | 1 | No | Yes | No need | 3.1% | 3.2% | 1.0% | 0 | Yes | Yes | Per 6 months | 0.7% | 3.2% | 2.5% | 360 | 11 (4.4) | 100 (39.1) | 137 (54.4) | 252 |
|  | 2 | Yes | No | Per 6 months | 0.7% | 5.8% | 0.2% | 120 | Yes | No | Per 3 months | 5.5% | 0.6% | 0.2% | 240 | 14 (5.6) | 117 (46.4) | 117 (46.4) | 252 |
|  | 3 | Yes | No | Per 3 months | 5.5% | 0.6% | 1.0% | 120 | Yes | Yes | No need | 3.1% | 3.2% | 1.0% | 240 | 15 (5.9) | 128 (50.8) | 105 (41.7) | 252 |
|  | 4 | Yes | Yes | Per 1 month | 3.1% | 3.2% | 0.2% | 0 | Yes | No | No need | 0.7% | 8.4% | 0.2% | 360 | 15 (6.0) | 170 (67.5) | 63 (25.0) | 252 |
|  | 5 | No | No | Per 6 months | 3.1% | 5.8% | 1.8% | 360 | No | No | Per 3 months | 5.5% | 5.8% | 1.0% | 0 | 16 (6.3) | 139 (55.2) | 93 (36.9) | 252 |
|  | 6 | Yes | Yes | Per 1 month | 7.8% | 8.4% | 1.8% | 240 | No | Yes | Per 1 month | 7.8% | 5.8% | 1.8% | 120 | 30 (11.9) | 30 (11.9) | 187 (74.2) | 252 |
|  | 7 | No | No | No need | 5.5% | 0.6% | 1.8% | 360 | No | Yes | Per 3 months | 3.1% | 0.6% | 1.8% | 0 | 10 (4.0) | 30 (11.9) | 207 (82.1) | 252 |
|  | 8 | No | Yes | Per 3 months | 5.5% | 8.4% | 2.5% | 240 | No | Yes | Per 1 month | 7.8% | 8.4% | 2.5% | 120 | 26 (10.3) | 180 (71.4) | 41 (16.3) | 252 |
|  | Total^*^ |  |  |  |  |  |  |  |  |  |  |  |  |  |  | 137 (6.8) | 894 (44.3) | 909 (45.1) | 2016 |

BLK indicates block; SCE indicates scenario; ATD indicates antidote; F-D indicates food-drug interaction; MONI indicates the frequency of blood monitoring; BLD indicates the risk of major bleeding; STK indicates the risk of stroke or systemic embolism; AMI indicates the risk of acute myocardial infarction; COST indicates out-of-pocket cost.

* The total number is person-time. Take block 1 for example: there were 254 patients were allocated to block 1, thus, there were 254 * 8 person-times in 8 scenarios.
