# Supplementary material for: Importance of attributes and willingness to pay for oral anticoagulant therapy in patients with atrial fibrillation in China: A discrete choice experiment
Source: PLoS Med. 2021 Aug 26;18(8):e1003730. doi: 10.1371/journal.pmed.1003730 (PMC8432810; doi:10.1371/journal.pmed.1003730)
Supplement: S5 File — (DOCX) [file pmed.1003730.s005.docx]

**S5 File. Preference weights estimated by mixed logit regression model excluding patients who failed in the test scenario (n = 470)**

| Attribute | Crude β (95% CI) | P value^*^ | Adjusted β (95% CI)^#^ | P value^*^ |
| --- | --- | --- | --- | --- |
| Out-of-pocket cost | -0.0012 (-0.0015, -0.0008) | <0.001 | -0.0014 (-0.0018, -0.0010) | <0.001 |
| Risk of AMI | -0.88 (-1.12, -0.64) | <0.001 | -1.01 (-1.30, -0.72) | <0.001 |
| Risk of stroke or systemic embolism | -0.73 (-0.79, -0.66) | <0.001 | -0.85 (-0.95, -0.76) | <0.001 |
| Risk of bleeding | -0.62 (-0.68, -0.55) | <0.001 | -0.73 (-0.82, -0.63) | <0.001 |
| Food-drug interaction | -0.40 (-0.60, -0.20) | <0.001 | -0.67 (-0.92, -0.43) | <0.001 |
| Antidote | 0.44 (0.20, 0.68) | <0.001 | 0.36 (0.07, 0.65) | 0.01 |
| Frequency of blood monitoring | -0.29 (-0.35, -0.23) | <0.001 | -0.34 (-0.42, -0.27) | <0.001 |
| Model specification | Removed patients failed in test scenario (crude model): Log likelihood = -1993; McFadden Pseudo R2 = 0.1988 | | | |
|  | Removed patients failed in test scenario (adjusted model): Log likelihood = -1114; McFadden Pseudo R2 = 0.2424 | | | |

β indicates coefficient and represents relative weight; negative value indicates negative preference. AMI indicates acute myocardial infarction.

* P values for coefficients were obtained by Wald test.

# Adjusted by age, sex, education level, income level, city, self-evaluated health score, history of cardiovascular disease/other vascular disease/any stroke/any bleeding, use of anticoagulant/antiplatelet; the correlation between any pair of attributes also involved in the model.
