# Supplementary material for: Importance of attributes and willingness to pay for oral anticoagulant therapy in patients with atrial fibrillation in China: A discrete choice experiment
Source: PLoS Med. 2021 Aug 26;18(8):e1003730. doi: 10.1371/journal.pmed.1003730 (PMC8432810; doi:10.1371/journal.pmed.1003730)
Supplement: S6 File — (DOCX) [file pmed.1003730.s006.docx]

**S6 File. Preference weights estimated by mixed logit regression model with frequency of blood monitoring as a categorical variable (n = 506)**

| Attribute | Crude β (95% CI) | P value^*^ | Adjusted β (95% CI)^#^ | P value^*^ |
| --- | --- | --- | --- | --- |
| Out-of-pocket cost | -0.0015 (-0.0018, -0.0011) | <0.001 | -0.0019 (-0.0023, -0.0014) | <0.001 |
| Risk of AMI | -0.90 (-1.16, -0.64) | <0.001 | -1.10 (-1.42, -0.77) | <0.001 |
| Risk of stroke or systemic embolism | -0.64 (-0.72, -0.56) | <0.001 | -0.72 (-0.82, -0.62) | <0.001 |
| Risk of bleeding | -0.52 (-0.60, -0.45) | <0.001 | -0.59 (-0.69, -0.49) | <0.001 |
| Food-drug interaction | -0.35 (-0.57, -0.13) | 0.002 | -0.62 (-0.91, -0.33) | <0.001 |
| Antidote | 0.56 (0.30, 0.82) | <0.001 | 0.55 (0.26, 0.84) | <0.001 |
| Frequency of blood monitoring | - | - | - | - |
| No need | Ref | Ref | Ref | Ref |
| Every 6 months | 0.14 (-0.07, 0.35) | 0.19 | 0.43 (0.18, 0.69) | <0.001 |
| Every 3 months | -0.37 (-0.57, -0.17) | <0.001 | -0.30 (-0.54, -0.05) | 0.02 |
| Every 1 month | -0.90 (-1.08, -0.71) | <0.001 | -0.85 (-1.09, -0.61) | <0.001 |
| Model specification | Monitoring as a categorical variable (crude model): Log likelihood = -2066; McFadden Pseudo R^2^ = 0.1956 | | | |
|  | Monitoring as a categorical variable (adjusted model): Log likelihood = -1990; McFadden Pseudo R^2^ = 0.2251 | | | |

β indicates coefficient and represents relative weight; negative value indicates negative preference. AMI indicates acute myocardial infarction.

* P values for coefficients were obtained by Wald test.

# Adjusted by age, sex, education level, income level, city, self-evaluated health score, history of cardiovascular disease/other vascular disease/any stroke/any bleeding, and use of anticoagulant/antiplatelet; the correlation between any pair of attributes also involved in the model.
