# Supplementary material for: Importance of attributes and willingness to pay for oral anticoagulant therapy in patients with atrial fibrillation in China: A discrete choice experiment
Source: PLoS Med. 2021 Aug 26;18(8):e1003730. doi: 10.1371/journal.pmed.1003730 (PMC8432810; doi:10.1371/journal.pmed.1003730)
Supplement: S7 File — (DOCX) [file pmed.1003730.s007.docx]

**S7 File. Preference weights estimated by mixed logit regression model with blocks of scenarios being included for adjustment (n = 506)**

| Attribute | Crude β (95% CI) | P value^*^ | Adjusted β (95% CI)^#^ | P value^*^ |
| --- | --- | --- | --- | --- |
| Out-of-pocket cost | -0.0011  (-0.0014, -0.0008) | <0.001 | -0.0013 (-0.0016, -0.0009) | <0.001 |
| Risk of AMI | -0.85 (-1.08, -0.62) | <0.001 | -0.96 (-1.27, -0.64) | <0.001 |
| Risk of stroke or systemic embolism | -0.70 (-0.76, -0.64) | <0.001 | -0.80 (-0.89, -0.71) | <0.001 |
| Risk of bleeding | -0.58 (-0.65, -0.52) | <0.001 | -0.67 (-0.77, -0.57) | <0.001 |
| Food-drug interaction | -0.36 (-0.55, -0.16) | <0.001 | -0.55 (-0.83, -0.28) | <0.001 |
| Antidote | 0.45 (0.22, 0.68) | <0.001 | 0.42 (0.16, 0.68) | 0.002 |
| Frequency of blood monitoring | -0.28 (-0.34, -0.22) | <0.001 | -0.31 (-0.39, -0.24) | <0.001 |
| Model specification | No adjustment for variables (crude model): Log likelihood = -2081; McFadden Pseudo R^2^ = 0.1898 | | | |
|  | With extra adjustment for monitoring (adjusted model): Log likelihood = -2029; McFadden Pseudo R^2^ = 0.2101 | | | |

β indicates coefficient and represents relative weight; negative value indicates negative preference. AMI indicates acute myocardial infarction.

* P values for coefficients were obtained by Wald test.

# Adjusted by blocks of scenarios, age, sex, education level, income level, city, self-evaluated health score, history of cardiovascular disease/other vascular disease/any stroke/any bleeding, and use of anticoagulant/antiplatelet. The correlation between any pair of attributes also involved in the model.
