# Supplementary material for: Importance of attributes and willingness to pay for oral anticoagulant therapy in patients with atrial fibrillation in China: A discrete choice experiment
Source: PLoS Med. 2021 Aug 26;18(8):e1003730. doi: 10.1371/journal.pmed.1003730 (PMC8432810; doi:10.1371/journal.pmed.1003730)
Supplement: S8 File — (DOCX) [file pmed.1003730.s008.docx]

**S8 File. Preference weights estimated by mixed logit regression model stratified by outpatient and inpatient setting (n = 504)^*^**

|  | Outpatient setting (n = 138) | | | | Inpatient setting (n = 366) | | | |
| --- | --- | --- | --- | --- | --- | --- | --- | --- |
|  | Crude β (95%CI) | P value^#^ | Adjusted β (95%CI)^&^ | P value^#^ | Crude β (95%CI) | P value^#^ | Adjusted β (95%CI) ^&^ | P value^#^ |
| Out-of-pocket cost | -0.0012  (-0.0018,  -0.0005) | <0.001 | -0.0016  (-0.0024,  -0.0008) | <0.001 | -0.0012  (-0.0016,  -0.0008) | <0.001 | -0.0014  (-0.0019,  -0.0010) | <0.001 |
| Risk of AMI | -0.95  (-1.41, -0.49) | <0.001 | -1.09  (-1.77, -0.42) | 0.001 | -0.80  (-1.08, -0.52) | <0.001 | -0.85  (-1.18, -0.52) | <0.001 |
| Risk of stroke or systemic embolism | -0.68  (-0.80, -0.55) | <0.001 | -0.90  (-1.12, -0.68) | <0.001 | -0.72  (-0.80, -0.65) | <0.001 | -0.85  (-0.95, -0.74) | <0.001 |
| Risk of bleeding | -0.59  (-0.73, -0.46) | <0.001 | -0.78  (-1.01, -0.55) | <0.001 | -0.60  (-0.68, -0.52) | <0.001 | -0.69  (-0.80, -0.58) | <0.001 |
| Food-drug interaction | -0.26  (-0.66, 0.14) | 0.20 | -0.68  (-1.26, -0.10) | 0.02 | -0.38  (-0.62, -0.14) | 0.002 | -0.67  (-0.96, -0.38) | <0.001 |
| Antidote | 0.63  (0.19, 1.08) | 0.005 | 0.42  (-0.23, 1.06) | 0.20 | 0.39  (0.10, 0.67) | 0.007 | 0.16  (-0.18, 0.50) | 0.35 |
| Frequency of blood monitoring | -0.28  (-0.39, -0.16) | <0.001 | -0.34  (-0.51, -0.17) | <0.001 | -0.28  (-0.35, -0.21) | <0.001 | -0.33  (-0.41, -0.24) | <0.001 |
| Model specification | Outpatient setting (crude model): Log likelihood = -557; McFadden Pseudo R^2^ = 0.1891 | | | | | | | |
|  | Outpatient setting (adjusted model): Log likelihood = -535; McFadden Pseudo R^2^ = 0.2219 | | | | | | | |
|  | Inpatient setting (crude model): Log likelihood = -1510; McFadden Pseudo R^2^ = 0.1926 | | | | | | | |
|  | Inpatient setting (adjusted model): Log likelihood = -1467; McFadden Pseudo R^2^ = 0.2154 | | | | | | | |

β indicates coefficient and represents relative weight; negative value indicates negative preference. AMI indicates acute myocardial infarction.

* There were 2 patients where their treatment settings were unknown.

# P values for coefficients were obtained by Wald test.

& Adjusted by age, sex, education level, income level, city, self-evaluated health score, history of cardiovascular disease/other vascular disease/any stroke/any bleeding, and use of anticoagulant/antiplatelet; the correlation between any pair of attributes also involved in the model.
