# Supplementary material for: Importance of attributes and willingness to pay for oral anticoagulant therapy in patients with atrial fibrillation in China: A discrete choice experiment
Source: PLoS Med. 2021 Aug 26;18(8):e1003730. doi: 10.1371/journal.pmed.1003730 (PMC8432810; doi:10.1371/journal.pmed.1003730)
Supplement: S9 File — (DOCX) [file pmed.1003730.s009.docx]

**S9 File. Preference weights estimated by mixed logit regression model with the exclusion of 28 patients who did not have any medical insurance (n = 478)**

|  | Crude β (95%CI) | P value^*^ | Adjusted β (95%CI)^#^ | P value^*^ |
| --- | --- | --- | --- | --- |
| Out-of-pocket cost | -0.0008 (-0.0011, -0.0005) | <0.001 | -0.0010 (-0.0013, -0.0006) | <0.001 |
| Risk of AMI | -0.90 (-1.14, -0.66) | <0.001 | -1.06 (-1.34, -0.78) | <0.001 |
| Risk of stroke or systemic embolism | -0.74 (-0.81, -0.68) | <0.001 | -0.86 (-0.95, -0.76) | <0.001 |
| Risk of bleeding | -0.62 (-0.69, -0.55) | <0.001 | -0.73 (-0.83, -0.63) | <0.001 |
| Food-drug interaction | -0.37 (-0.57, -0.16) | <0.001 | -0.64 (-0.90, -0.38) | <0.001 |
| Antidote | 0.51 (0.27, 0.75) | <0.001 | 0.46 (0.20, 0.73) | <0.001 |
| Frequency of blood monitoring | -0.30 (-0.36, -0.24) | <0.001 | -0.33 (-0.41, -0.26) | <0.001 |
| Model specification | Patients who had medical insurance (crude model): Log likelihood = -1940; McFadden Pseudo R2 = 0.1996 | | | |
|  | Patients who had medical insurance (adjusted model): Log likelihood = -1890; McFadden Pseudo R2 = 0.2203 | | | |

β indicates coefficient and represents relative weight; negative value indicates negative preference. AMI indicates acute myocardial infarction.

* P values for coefficients were obtained by Wald test.

# Adjusted by age, sex, education level, income level, city, self-evaluated health score, history of cardiovascular disease/other vascular disease/any stroke/any bleeding, and use of anticoagulant/antiplatelet; the correlation between any pair of attributes also involved in the model.
