# Supplementary material for: Importance of attributes and willingness to pay for oral anticoagulant therapy in patients with atrial fibrillation in China: A discrete choice experiment
Source: PLoS Med. 2021 Aug 26;18(8):e1003730. doi: 10.1371/journal.pmed.1003730 (PMC8432810; doi:10.1371/journal.pmed.1003730)
Supplement: S10 File — (DOCX) [file pmed.1003730.s010.docx]

**S10 File. Preference weights estimated by nested logit model with opt-out option included (n = 506)**

| Attribute | Crude β (95% CI) | P value^*^ | Adjusted β (95% CI)^#^ | P value^*^ |
| --- | --- | --- | --- | --- |
| Out-of-pocket cost | -0.0010 (-0.0013, -0.0007) | <0.001 | -0.0010 (-0.0013, -0.0007) | <0.001 |
| Risk of AMI | -0.85 (-1.06, -0.64) | <0.001 | -0.85 (-1.06, -0.64) | <0.001 |
| Risk of stroke or systemic embolism | -0.64 (-0.70, -0.59) | <0.001 | -0.64 (-0.70, -0.59) | <0.001 |
| Risk of bleeding | -0.57 (-0.63, -0.51) | <0.001 | -0.57 (-0.64, -0.51) | <0.001 |
| Food-drug interaction | -0.46 (-0.62, -0.29) | <0.001 | -0.45 (-0.62, -0.28) | <0.001 |
| Antidote | 0.45 (0.24, 0.66) | <0.001 | 0.45 (0.23, 0.67) | <0.001 |
| Frequency of blood monitoring | -0.27 (-0.32, -0.22) | <0.001 | -0.27 (-0.32, -0.22) | <0.001 |
| Model specification | With opt-out option included (crude model): Log likelihood = -3047; McFadden Pseudo R^2^ = 0.1299 | | | |
|  | With opt-out option included (adjusted model): Log likelihood = -2906; McFadden Pseudo R^2^ = 0.1703 | | | |

β indicates coefficient and represents relative weight; negative value indicates negative preference. AMI indicates acute myocardial infarction.

* P values for coefficients were obtained by Wald test.

# Adjusted by age, sex, education level, income level, city, self-evaluated health score, history of cardiovascular disease/other vascular disease/any stroke/any bleeding, and use of anticoagulant/antiplatelet.
