# Supplementary material for: Importance of attributes and willingness to pay for oral anticoagulant therapy in patients with atrial fibrillation in China: A discrete choice experiment
Source: PLoS Med. 2021 Aug 26;18(8):e1003730. doi: 10.1371/journal.pmed.1003730 (PMC8432810; doi:10.1371/journal.pmed.1003730)
Supplement: S11 File — (DOCX) [file pmed.1003730.s011.docx]

**S11 File. Preference weights estimated by multinomial logit model with opt-out option included (n = 506)**

| Attribute | Crude β (95% CI) | P value^*^ | Adjusted β (95% CI)^#^ | P value^*^ |
| --- | --- | --- | --- | --- |
| Out-of-pocket cost | -0.0009 (-0.0012, -0.0007) | <0.001 | -0.0009 (-0.0012, -0.0007) | <0.001 |
| Risk of AMI | 0.26 (0.15, 0.36) | <0.001 | 0.22 (0.12, 0.33) | <0.001 |
| Risk of stroke or systemic embolism | -0.33 (-0.36, -0.30) | <0.001 | -0.34 (-0.37, -0.31) | <0.001 |
| Risk of bleeding | -0.20 (-0.22, -0.17) | <0.001 | -0.21 (-0.24, -0.18) | <0.001 |
| Food-drug interaction | 0.30 (0.19, 0.42) | <0.001 | 0.28 (0.17, 0.40) | <0.001 |
| Antidote | -0.23 (-0.38, -0.08) | 0.002 | -0.21 (-0.36, -0.06) | 0.006 |
| Frequency of blood monitoring | -0.21 (-0.26, -0.17) | <0.001 | -0.21 (-0.26, -0.17) | <0.001 |
| Model specification | With opt-out option included (crude model): Log likelihood = -3181; McFadden Pseudo R^2^ = 0.0917 | | | |
|  | With opt-out option included (adjusted model): Log likelihood = -3031; McFadden Pseudo R^2^ = 0.1344 | | | |

β indicates coefficient and represents relative weight; negative value indicates negative preference. AMI indicates acute myocardial infarction.

* P values for coefficients were obtained by Wald test.

# Adjusted by age, sex, education level, income level, city, self-evaluated health score, history of cardiovascular disease/other vascular disease/any stroke/any bleeding, and use of anticoagulant/antiplatelet.
