# Supplementary material for: Importance of attributes and willingness to pay for oral anticoagulant therapy in patients with atrial fibrillation in China: A discrete choice experiment
Source: PLoS Med. 2021 Aug 26;18(8):e1003730. doi: 10.1371/journal.pmed.1003730 (PMC8432810; doi:10.1371/journal.pmed.1003730)
Supplement: Attachment — Submitted filename: response to PMEDICINE-D-21-00677R2.pdf [file pmed.1003730.s013.pdf]

Beryne Odeny, PhD

Associate Editor, *PLOS Medicine*

30 June, 2021

Dear Dr. Beryne Odeny,

RE: Manuscript ID PMEDICINE-D-21-00677R2 – "Importance of attributes and willingness-to-pay for oral anticoagulant therapy in patients with atrial fibrillation in China: A discrete choice experiment": response to the editors' and reviewers' comments.

Thank you for the comments in the recent decision email dated 23 June 2021. We appreciate your consideration of publishing this paper in the journal after the remaining issues are addressed. Our responses to the editors' and reviewers' comments are given point-by-point below in red.

Thank you again for your consideration.

Best wishes,

Esther W. Chan, PhD

Associate Professor and Research Lead, Centre for Safe Medication Practice and Research
Department of Pharmacology and Pharmacy, Li Ka Shing Faculty of Medicine, The University of Hong Kong
Associate Professor, The University of Hong Kong Shenzhen Institute of Research and Innovation, Shenzhen, China

1/F, Jockey Club Building for Interdisciplinary Research, Li Ka Shing Faculty of Medicine, 5 Sassoon Road, Pokfulam, Hong Kong SAR, China

Tel: +852-28315110; Fax:+852-28170859; Email: ewchan@hku.hk

Requests from the editors:

Thank your responses. Please address, in sufficient detail, the following outstanding points before we proceed:

1. In the manuscript text, please indicate: (1) that the analysis was prespecified and the specific hypotheses you intended to test, (2) the analytical methods by which you planned to test them, (3) the analyses you actually performed, and (4) when reported analyses differ from those that were planned, transparent explanations for differences that affect the reliability of the study's results. If a reported analysis was performed based on an interesting but unanticipated pattern in the data, please be clear that the analysis was data-driven.

Thank you for your suggestions. We have added several sentences in the main text to reflect the approach taken for the planning of the analysis, the analysis and reporting (Lines 272-276 in the tracked version).

2. Please define the all abbreviations in footnote for tables and/or Figures e.g. AMI, RMB1, mWTP and so forth.

We have defined all abbreviations of AMI, RMB, mWTP in the footnote of the tables in both main text and supplementary files. (Under tables 1-4 in main text; under tables in supplementary file 1-3 and 5.)

Comments from the reviewers:

Reviewer #2: Many thanks authors for their great effort to improve the manuscript. All my questions/comments were professionally addressed. I am satisfied with the response and revision. No further issues needing attention.

Thank you for your time and suggestions in the review process.

Reviewer #3: All my inquiries and comments on the previous version have been answered, or authors justified their approach if they could not provide readers with further data. Thank you.

Thank you for your time and suggestions in the review process.

Reviewer #4: Thanks to the authors for revising the manuscript and responding my questions, I have no further comments.

Thank you for your time and suggestions in the review process.

Any attachments provided with reviews can be seen via the following link:
****

In compliance with data protection regulations, you may request that we remove your personal registration details at any time. (Use the following URL: https://www.editorialmanager.com/pmedicine/login.asp?a=r). Please contact the publication office if you have any questions.
