# Supplementary material for: Importance of attributes and willingness to pay for oral anticoagulant therapy in patients with atrial fibrillation in China: A discrete choice experiment
Source: PLoS Med. 2021 Aug 26;18(8):e1003730. doi: 10.1371/journal.pmed.1003730 (PMC8432810; doi:10.1371/journal.pmed.1003730)
Supplement: Attachment — Submitted filename: response to PMEDICINE-D-21-00677R1.docx [file pmed.1003730.s012.docx]

20 May 2021

Beryne Odeny, PhD

Associate Editor, *PLOS Medicine*

Dear Dr. Beryne Odeny,

**RE: Manuscript ID PMEDICINE-D-21-00677R1 – “Understanding oral anticoagulant preferences of patients with atrial fibrillation in China and willingness-to-pay: Evidence from a discrete choice experiment”: response to the editors’ and reviewers’ comments for the manuscript.**

Thank you very much for the comments in the recent decision email dated 30 April 2021. We appreciate this opportunity to further revise our manuscript. Our responses to the editors’ and reviewers’ comments are given point-by-point below in red.

**Esther W. Chan, PhD**

Associate Professor and Research Lead, Centre for Safe Medication Practice and Research, Department of Pharmacology and Pharmacy, Li Ka Shing Faculty of Medicine, The University of Hong Kong

Associate Professor, The University of Hong Kong Shenzhen Institute of Research and Innovation, Shenzhen, China

1/F, Jockey Club Building for Interdisciplinary Research, Li Ka Shing Faculty of Medicine, 5 Sassoon Road, Pokfulam, Hong Kong SAR, China

Tel: +852-28315110; Fax:+852-28170859; Email: ewchan@hku.hk

-----------------------------------------------------------
Requests from the editors:

• Please revise your title according to PLOS Medicine's style. Your title must be nondeclarative and not a question. It should begin with main concept if possible. Please place the study design ("A discrete choice experiment,") in the subtitle (i.e., after a colon).

Thank you for the comment. We changed our title to “Importance of attributes and willingness-to-pay for oral anticoagulant therapy in patients with atrial fibrillation in China: A discrete choice experiment” (Lines 3-4 of tracked version).

• The Data Availability Statement (DAS) requires revision. For each data source used in your study:
a) If the data are owned by a third party but freely available upon request, please note this and state the owner of the data set and contact information for data requests (web or email address). Note that a study author cannot be the contact person for the data.
b) If the data are not freely available, please include an appropriate contact (web or email address) for inquiries (again, this cannot be a study author).

Thank you for the comment. The data we used for this study could not be considered as either case a or case b. Since the data used originate from surveys collected from individual participants recruited for this study, the raw un-anonymized data is not available due to ethical restrictions, whereas all anonymized data has been summarized and presented in the manuscript and supplementary materials. As such, the DAS should be “As the data was collected via a questionnaire survey, due to the nature of ethical restriction in this study, participants of this study did not agree for their un-anonymized data to be shared publicly. Authors of this article confirm that all anonymized data supporting the findings of this study are available within the article and its supplementary materials.”

• In the abstract Methods and Findings:
- Please structure your abstract using the PLOS Medicine headings (Background, Methods and Findings, Conclusions). Please combine the Methods and Findings sections into one section, “Methods and findings”.
-Please ensure that all numbers presented in the abstract are present and identical to numbers presented in the main manuscript text.
- Please quantify the main results with both 95% CIs and p values.
- Please include the important dependent variables that are adjusted for in the analyses.
- In the last sentence of the Abstract Methods and Findings section, please describe the main limitation(s) of the study's methodology.

Thank you for the comment. The abstract has been revised (lines 37-73 of tracked version).

• Did your study have a prospective protocol or analysis plan? Please state this (either way) early in the Methods section.
a) If a prospective analysis plan (from your funding proposal, IRB or other ethics committee submission, study protocol, or other planning document written before analyzing the data) was used in designing the study, please include the relevant prospectively written document with your revised manuscript as a Supporting Information file to be published alongside your study, and cite it in the Methods section. A legend for this file should be included at the end of your manuscript.
b) If no such document exists, please make sure that the Methods section transparently describes when analyses were planned, and when/why any data-driven changes to analyses took place.
c) In either case, changes in the analysis-- including those made in response to peer review comments-- should be identified as such in the Methods section of the paper, with rationale.

Thank you for the comment. We did not have a prospective protocol, as this is an exploratory cross-sectional study. Patient recruitment in Shenzhen site was terminated due to the COVID-19 pandemic in early 2020 (in table 2 footnote). The main analysis was conducted as being planned in the beginning of this study. Sensitivity analyses were designed and conducted to test the robustness of this study after results were obtained from the main analysis.

• In statistical methods, please refer to any post-hoc corrections to correct for multiple comparisons during your statistical analyses. If these were not performed please justify the reasons. Please refer to our statistical reporting guidelines for assistance ()

Thank you for the comment. The objective of this study is to evaluate patients’ preference on attributes of oral anticoagulants. Regression models were used for the estimations. The issues of multiple comparisons are not applicable in our study.

• Please describe how you selected your adjustment variables.

Thank you for the comment. We have revised (Lines 272-277).

• In the Methods and Results section:
- Please provide both adjusted and unadjusted estimates. In all tables and supplements, please provide the unadjusted comparisons for the adjusted comparisons
- Please provide 95% CIs and p values for estimates. Include in both tables and main text
- When a p value is given, please specify the statistical test used to determine it.

Thank you for the comments. All crude and adjusted estimations with 95% CI for coefficients of attributes have been presented in tables. All p values have been presented in tables except for mWTP, as Krinsky and Robb’s method were used for 95% CI calculation for mWTP. This method uses a bootstrapping approach to obtain the empirical distribution of each of the mWTP from 10,000 sets of replicated coefficients, and 95% CI for each of the mWTP is identified on the basis of each empirical distribution(1). As there is no standardized probability density could be obtained from this approach, it is hard to calculate p value without a z-score or t-score.

• The term "trend" is used to refer to a nonsignificant P value. The term trend should be used only when the test for trend has been conducted. Please revise accordingly.

Thank you for the comment. We have replaced “trends” with “patterns” (Line 334 of tracked version).

• The terms gender and sex are not interchangeable (as discussed in http://www.who.int/gender/whatisgender/en/ ); please use the appropriate term.

Thank you for the comment. We used the biologically defined term “sex”, which is accepted by all recruited patients in this study.

• Please refer to high income countries rather than "Western" countries.

Thank you for the comment. We have replaced “western countries” with “high-income countries” (Lines 178, 397 of tracked version).

• Please replace “warfarin users” with “patients who use warfarin drugs.”

Thank you for the comment. We have replaced “warfarin users” with “patients who use warfarin drugs” (Line 217 of tracked version).

• Please use the "Vancouver" style for reference formatting and see our website for other reference guidelines <https://journals.plos.org/plosmedicine/s/submission-guidelines#loc-references>.

Thank you for the comment. We have revised.

• Please define the abbreviations in tables and/or Figures e.g. AMI, RMB
Please include line numbers in your next draft.

Thank you for the comment. We have added a list of abbreviations after the abstract, and line numbers (Lines 122-141 of tracked version).

Reference

1.	Aizaki H. support.CEs: Basic functions for supporting an implementation of choice experiments. R package version 0.5-0. 2021.

Comments from the reviewers:

Reviewer #1: Understanding oral anticoagulant preferences of patients with AF in China and willingness-to-pay

This is a well-written report of a rigorously conducted conjoint analysis study of patient choice factors related to OACs in China. longitudinal study of student learning about "EBM" in a pharmacy school. Questions and suggestions whose responses could strengthen the manuscript are provided below. Relatively minor grammatical copyediting is required.

Introduction

Well-written and justified.

Thank you for your recognition.

Methods

Choice of the 7 attributes: It is stated these were chosen based on literature search and "ranking by an expert panel with forty cardiologists and forty eligible patients", but the supplement doesn't disclose the list from which the 7 were chosen. More disclosure about this seems necessary since readers may wonder why some attributes were or were not chosen. For example, "risk of non-fatal acute myocardial infarction" is questionable as a risk of NOAC treatment. This was initially identified as a possible risk with dabigatran, but largely clarified subsequently as likely not a drug-attributable risk. "Risk of non-fatal stroke (either ischemic or hemorrhagic)" contains a dichotomy... one is an efficacy attribute of the drugs, and one is a toxicity attribute. There is an inherent trade-off between ischemic and hemorrhagic stroke between OACs and it is misleading to include both in a single attribute. Justify.

Thank you for your comment. Fifteen attributes of oral anticoagulants, which might influence patients’ decision making during anticoagulation therapy, were ranked by 40 patients and 40 clinicians. They are: fatal bleeding risk, non-fatal major bleeding risk, non-fatal minor bleeding risk, non-fatal any bleeding risk, fatal stroke or MI risk, non-fatal major stroke risk, non-fatal minor stroke risk, non-fatal any stroke risk, non-fatal MI risk, antidote, blood test requirement, food-drug interaction, dose adjustment, frequency of drug administration, and out-of-pocket cost. Risks of bleeding, stroke and MI were the most concerned attributes for both patients and clinicians. However, we found that patients’ decisions were extremely dominated by fatal risks in our exploratory experiment. We decided to remove all fatal risks. We also found that patients had difficulties in distinguishing ischemic stroke and hemorrhagic stroke. We decided to use these two terms together to mainly assess their preference on stroke.

Patients in China may have concerns about other risks during the treatment for AF, especially for the risk related to heart, for example the risk of MI. There is also one US study which included risk of MI as one of the attribute(1). Therefore, we selected these 7 attributes for this study. We have added paragraphs in footnote of table 1 to interpret our attributes selection strategy in details.

Profiles/scenarios were used instead of individual-attribute tradeoffs for sample-size and participant burden reasons, and "names of alternatives could distract patients' attention from the targeting attributes, and bias their preferences" were justifiable performed. However, this implies that the scenarios corresponded to attributes of actual drugs. If so, the Results and Discussion should "unmask" these so more meaningful discussion about the actual therapeutic implications of the results is possible. If not, why does the Limitations section say, "we do not know whether patients prefer NOACs or warfarin due to the unlabelled design".

Thank you for the question. Those 7 chosen attributes are the most important factors that patients might mainly rely on when making decision for their anticoagulation therapy. In other words, these 7 attributes might represent the most frequently encountered profiles of actual drugs. We admit that there might be some scenarios in where the combinations of all the levels of attributes could just represent either NOACs or warfarin. However, it is also possible that a combination of attributes with different levels, for example, had food-drug interaction, which is the characteristics of warfarin, but no need for blood monitoring, which is the characteristics of NOACs. In this case, it is hard to say the alternative represent warfarin or NOACs. Furthermore, the actual NOACs or warfarin may have other “hidden” attributes they were not included in our study. Patients might take these “hidden” attributes into consideration if labeled design was used. Since our study used “unlabeled design”, in order to avoid over interpreting, we said “we cannot fully ascertain whether patients prefer NOACs or warfarin due to the unlabeled design”.

Results

Well-written. No specific comments.

Thank you for your recognition.

Discussion

The authors do a good job of interpreting the results in the context of Chinese culture.

"...we included the 7 most important attributes on the basis of the literature review and pilot study" - see above comments and juitfiy or modify this statement.

Thank you for your comment. As stated in previous response, we have added paragraphs in footnote of table 1 to interpret our attributes selection strategy in details. We have also revised the sentence in the main text (Lines 435-437 of tracked version).

Conclusion

"...better treatment." Clarify what is meant by "better" here. More precision is required.

Thank you for your comment. The conclusion has been revised (Lines 72-73, 458-459 of tracked version).

Reference

1.	Najafzadeh M, Gagne JJ, Choudhry NK, Polinski JM, Avorn J, Schneeweiss SS. Patients' preferences in anticoagulant therapy: discrete choice experiment. Circ Cardiovasc Qual Outcomes. 2014;7(6):912-9.

Reviewer #2: This is an interesting and useful study on the oral anticoagulant preferences of patients with atrial fibrillation in China using discrete choice experiment (DCE) design. However, there are a few major issues needing attention.

1) Study design. There seems to be some inconsistencies between study design, conduct and analysis. The DCE design includes 7 attributes with up to 4 levels which leads to 4096 hypothetical profiles for each alternative which is a lot. It said in the methods "16 scenarios were randomly divided into two blocks with 8 for each. Eligible patients were also randomly assigned to two blocks". It's very confusing as all the sample size and analyses later on were based on 8 scenarios. Should stick to 16 or 8 scenarios if designed so, but designed 16 scenarios then reduced to 8 by randomised into tow blocks? Why not started with 8 or merge the 16 into 8? Any bias introduced? Any adjustment in the analyses? Because the patients in different blocks will have different 8 scenarios and then how come to analyse them together?

Thank you for the question. As the number of attributes and levels is large, using too few scenarios (combinations of attributes with different levels) would make it difficult to take full advantage of all levels of attributes (some levels of certain attributes may even not exist in the scenarios), and thus hard to precisely assess the impact of attributes. Sixteen is the minimum number of scenarios which can take advantage of all levels of attributes in our study. All of these 16 scenarios could be distributed to each patient theoretically. However, considering practical issues while the surveys are being filled out by each patient, having to decide for 16 scenarios would reduce the response rate and quality. Therefore, we randomly divided those 16 scenarios into two blocks to give each patient an acceptable workload.

Sample size calculation is based on the number of scenarios presented to each patient (i.e. 8 scenarios). We have revised the statement for scenarios allocation in the manuscript to make it clearer and avoid confusion. Dividing a large number of scenarios into several small blocks is an approach which has also been used in other DCE studies(1-4). The main outcome in mixed logit regression model in our study is whether a certain alternative is chosen. Exposures are the attributes of alternatives. One of the assumptions of analysis for DCE using mixed logit regression model is that, there is a linear relationship between attributes and the log-odds of a certain alternative being chosen. One unit or level changes in a certain attribute will induce a certain change in the probability of a certain alternative being chosen. The only difference between the two blocks in the DCE design in this study is the levels of attributes of the scenarios. With randomization, the distribution of levels of attributes across the two blocks are similar (supplementary 3). Thus, patients allocated to two blocks faced scenarios with similar variation.

In our main analysis, we adjusted the potential effect from interactions between any pairs of different attributes. We also included a sensitivity analysis in the supplementary 7 to adjust for the block assigned to the patients. The results are consistent to that in our main analysis.

2) Again, the DCE was designed with 16 scenarios and 3 alternatives. But both the sample size and main analyses were based on 2 alternatives (logit regression) by exculding the 'opt-out' option. Why? and what's the point of designing 3 alternatives?

Thank you. The third alternative is an opt-out option. Levels of its attributes are all fixed. Patients will choose opt-out only if they don’t want to choose drug A nor drug B. Including an opt-out option can provide a more accurate estimation on the preference for each attribute, in other words, it can reveal the true preference, without forcing patients to randomly choose A or B if they do not have a preference for either drug(5). The sample size calculation was based on the number of alternatives with variated attribute levels. As all the attribute levels of opt-out option are fixed, this option does not contribute to sample size calculation(6). We used a mixed logit regression model for the main analysis. One of the most important assumption of this model is that outcomes should be independent. In other words, outcomes (i.e. the probability of choosing alternatives) should only be influenced by explanatory variables (attributes). However, decision of choosing “opt-out” highly depends on drug A and drug B. Patients chose “opt-out” only if they did not choose drug A nor drug B. Considering the violation of independent assumption, those scenarios with “opt-out” option being selected were excluded from the main analysis. We conducted a sensitivity analysis using a nested logit model to account for the dependent outcome issue from the “opt-out” option(7). The results were consistent with main analysis (supplementary 10). We used the term “option” instead of “alternative” for opt-out to make it clearer (Lines 309-310 of tracked version).

3) Sample size. Sample size was based on 2 alternatives (excluding opt-out) and 8 scenarios and maximum 4 levels for attributes, but actually it's 3 alternatives and 16 scenarios. Does it mean the current sample size of around 500 patients is under powered?

Thank you. According to Orme’s rule of thumb formula, minimum sample size = 500 * largest level of attributes / number of scenarios / number of non opt-out alternatives(6). In our study, we estimated the sample size based on the number of scenarios presented to each patient (i.e. 8 scenarios), which is a conservative estimation resulting in the largest minimum required sample size. In addition, as patients will only choose “opt-out” if they do not like drug A nor drug B, and all levels of “opt-out” are fixed, the “opt-out” does not contribute to sample size calculation. Therefore, our final sample size 506 is sufficient. We have revised sentences for sample size calculation to make it clearer (Lines 250-254 of tracked version).

4) Irrational respondent. It said in the method "Those who did not choose the dominated one were considered as irrational and were corrected by investigators before answering the remaining questions". It's inadequate to 'correct' irrational respondents but instead should identify and remove those irrational respondents. In table 1, need to report the both rational and irrational respondents within Beijing and Shenzhen columns.

Thank you for the comment. We admit “irrational” is not a proper term to describe those patients who did not choose the correct choice in the test scenario. Although these patients might not understand the questionnaire very well in the beginning, investigators immediately explained the meaning of levels of attributes to them. These patients were allowed to continue answering the questionnaire only when their understanding was confirmed by investigators. After the investigators’ explanation, these patients were considered to have similar understanding as other patients did and were included in analyses. To assess whether the responses from these patients would influence our results, we included a sensitivity analysis where these patients were excluded, and the results were consistent with the main analysis. We revised sentences with the word “irrational” to make it clearer, and reported the number of these patients from both Beijing and Shenzhen (Lines 245-248, and table 2 of tracked version).

5) Analysis. The main analyses were currently based on all patients. However, the irrational respondents should be removed, and the main analysis should focus on those rational respondents.

Thank you for the comment. As stated in the response for comment #4, we have already conducted a sensitivity analysis excluding these patients (supplementary 5). All results were consistent to our main one. We also revised sentences with “irrational” to make it clearer, and added number of these patients from both Beijing and Shenzhen (Lines 245-248, and table 2 of tracked version).

6) Again, I am not comfortable with the main analyses only on two alternatives by excluding 'opt-out', then why did authors design 3 alternatives with opt-out in the first place? The sensitivity analysis using multinomial
logistic regression by including 'opt-out' gave very different results to the current analyses was a bit worrying and needs further consideration and discussion on the study design and suitability of the main analyses on only two alternatives.

Thank you for the comment. As stated in the responses for comment #1-3, including opt-out option can reveal patients’ true preference and avoid forcing them to make decision randomly. Patients will choose opt-out option only if they do not like drug A nor drug B. Consequently by definition, the outcome of choosing opt-out is highly dependent on the outcome of choosing drug A and drug B.

Mixed logit regression, multinomial logit regression and conditional logistic regression are models which have been mainly used for DCE analysis in other studies. One of the most important assumption for these models is that outcomes should be independent. In other words, outcomes (i.e. the probability of choosing alternatives) should only be influenced by explanatory variables (attributes). However, decision of choosing “opt-out” highly depends on drug A and drug B. Patients chose “opt-out” only if they did not choose drug A nor drug B. The decision of choosing “opt-out” is not based on the evaluation on attributes levels among those three. We revised the sentence (Lines 267-269 of tracked version) to make it clearer.

In order to explore the impact of “opt-out” on our results, we used a multinomial logit model with the inclusion of opt-out (supplementary 11). The large discrepancy of results compared to our main results indicated mixed logit model and multinomial logit model are not suitable for DCE analysis if “opt-out” is included. We also used a nested logit model, which can deal with the issue of dependent outcomes (supplementary 10)(7). The results were consistent with main one. However, nested logit model cannot account for random effects. Since it is hard to obtain all patient-related information in a cross-sectional study using questionnaire survey, there might be still some unobserved confounders. The mixed logit regression model can take this into consideration. In our study, controlling random effects is more important than impact from “opt-out”. Thus, we chose mixed logit model as our primary statistical analysis model. As different models have their own limitations, we could only use sensitivity analyses to test the robustness of our results.

7) Supplementary Table 1. Attributes and levels should appear in the main text as Table 1.

Thank you. We have moved supplementary table 1 to main text as table 1.

8) Generalisability of the finding. The two hospitals in China, the Chinese PLA General Hospital and HKU-Shenzhen Hospital, are top hospitals in China in two developed and affluent cities. Patients from these two hospital may not be representative of Chinese patients in general so need to discuss this in the limitation.

Thank you. We have added this part in limitation (Lines 451-453 of tracked version).


Reference

1.	Najafzadeh M, Gagne JJ, Choudhry NK, Polinski JM, Avorn J, Schneeweiss SS. Patients' preferences in anticoagulant therapy: discrete choice experiment. Circ Cardiovasc Qual Outcomes. 2014;7(6):912-9.
2.	Ghijben P, Lancsar E, Zavarsek S. Preferences for oral anticoagulants in atrial fibrillation: a best-best discrete choice experiment. Pharmacoeconomics. 2014;32(11):1115-27.

3.	Rozier MD, Ghaferi AA, Rose A, Simon NJ, Birkmeyer N, Prosser LA. Patient preferences for bariatric surgery: findings from a survey using discrete choice experiment methodology. JAMA Surg. 2019;154(1):e184375.

4.	Zanolini A, Sikombe K, Sikazwe I, Eshun-Wilson I, Somwe P, Bolton Moore C, et al. Understanding preferences for HIV care and treatment in Zambia: evidence from a discrete choice experiment among patients who have been lost to follow-up. PLoS Med. 2018;15(8):e1002636.
5.	Veldwijk J, Lambooij MS, de Bekker-Grob EW, Smit HA, de Wit GA. The effect of including an opt-out option in discrete choice experiments. PLoS One. 2014;9(11):e111805.

6.	Orme BK e. Sample size issues for conjoint analysis. Getting started with conjoint analysis: strategies for product design and pricing research (4th ed.). Madison, Wis: Research Publishers LLC. 2019.

7.	de Blaeij AT, Nunes PALD, van den Bergh JCJM. 'No-choice' options within a nested logit model: one model is insufficient. Applied Economics. 2007;39:1245-52.

Reviewer #3: General Comments
This study is generally well-designed. The quality of survey design, analysis, and the manuscript is good enough. Whether this study is sufficiently innovative (originality) needs a third-party evaluation.

Thank you for your recognition.

Potential edits for the entire manuscript: This is preference and WTP on specific attributes of oral anticoagulants, rather than the preference on specific anticoagulants. Consider clarification through the entire manuscript.

Thank you for the suggestion. We changed our title into “Importance of attributes and willingness-to-pay for oral anticoagulant therapy in patients with atrial fibrillation in China: A discrete choice experiment”, and rephrase sentences to emphasize the key point of this study is to evaluate patients’ preference on attributes of oral anticoagulants in context (Lines 3-4, 52, 78, 87-88, 92, 182 of tracked version).

Intro

Paragraph 1
"weighted prevalence" is unclear terminology. What are the factors that the prevalence calculation was weighted for?

Thank you for the comment. We have revised the sentence (Line 146 of tracked version).

Paragraph 2
Cost may or may not be considered as a preference factor. As a reviewer, I would love to classify this as an external determinant rather than preference. Are we able to replace this with "affordability" ?

Thank you for the comment. We have revised (Line 153 of tracked version).

This sentence is unclear: "Although NOACs captured 92% of the oral anticoagulant therapy market, only 28% of patients with AF in China were covered in 2017." Does this sentence mean that a 92% of anticoagulation management involves NOAC in China, or across the world, but the usage rate becomes 28% when the analysis is limited to the Chinese AF population?

Thank you for the question. We have revised the sentence (Lines 155-157 of tracked version).

This sentence is unclear: "30% of costs are derived from patients attending outpatient departments". Does this mean that 30% of the AF management cost is incurred at outpatient setting including outpatient pharmacy and clinic visits, and 70% came from inpatient management?

Thank you for the question. We have revised the sentence (Lines 158-159 of traced version).

Methods:
Patient recruitment from both outpatient and inpatient - see if there is a difference in the preference determined by the healthcare setting where patients were enrolled.

Thank you for the comment. We have added one sensitivity analysis stratifying patients by outpatient and inpatient setting (supplementary 8). The results are consistent with main analysis.

Introduction for DCE seems two long. Probably you do not need the first two sentences of the "Discrete choice Experiment" section.

Thank you for the suggestion. We have shortened the sentence for DCE explanation. Considering the readers research backgrounds might vary, we prefer to keep a brief sentence to describe what is DCE (Lines 167-170 of tracked version).

Discrete Choice Experiment
This is unclear: "If patients were unable to complete the investigation, their guardians were considered as a proxy." Does this mean that care provider/guardian consented to participate and completed the DCE survey? IF that is the case, probably the whole analysis needs to be stratified by the type of respondent - patients vs. care providers/guardians - or needs to exclude responses from caregivers/guardians.

Thank you for the comment. In China, it is common that patients’ family members or guardians visit clinics to obtain prescriptions for regular or chronic medications, particularly for older patients. We planned to distribute consent forms and questionnaires to those guardians and asked if the patients would like to participate in our survey. However, there was no such case in our investigation. All patients answer questions by themselves. To avoid confusion, we have deleted this sentence (Lines 210-211 of tracked version).

Attribute and level identification.
Minor comment: cite reference to every single number makes the manuscript less easy to follow. Probably cite at the end of the sentence.

Thank you for the suggestion. We have revised (Lines 221-223).

This probably is self evident, but want to make sure that "antidote" means there is an antidote for the case of adverse reaction ?

Thank you for the question. Yes, and we used “Antidote could be provided if there was a side effect when administrating oral anticoagulant” in table 1 (current version) to explain what is “antidote”.

Probably a short description on health literacy to understand the DCE questions would help.

Thank you for the suggestion. We have added one sentence in data collection section (Lines 259-260 of tracked version).

Results
Subgroup analysis:
If the authors can project the 95% confidence interval of the difference in WTP, that would improve the message.

Thank you for the suggestion. We agree with reviewer’s point that it would be informative if we could provide statistical tests on the difference of marginal WTP between subgroups, as 95% CI of some of the mWTP in our study were wide. However, due to the limitations in statistical designs and methodology of DCE, it is challenging for us to conduct such tests. Testing difference of mWTP between subgroups means that the subgroups would be considered as an exposure, mWTP would be considered as the outcome, and all the other factors should be balanced. One of the reasons that we did not consider conducting this test even if we could, is that, due to the nature of DCE study, it is hard to identify two groups with balanced characteristics according to the patient population, as well as their baseline information which we had. In addition, mWTP is actually a transformed ratio of the coefficient of a certain attributes to the coefficient of out-of-pocket cost in a statistical model(1). Testing the difference of mWTP implies testing the difference of coefficients of two different models. Whether this is equivalent to the difference testing on patients’ marginal willingness-to-pay still needs further discussion. Moreover, similar studies did not conduct the tests neither. We would like to provide findings to readers only if we are confident about them. Therefore, we prefer not to provide 95% CI of the difference in WTP in this study.


Discussion
the relative importance of blood monitoring compared to major bleeding in our study was 9-15 times higher than that in two western studies: Is this because of the access to healthcare facility? Or Shouldn't this be a matter?
It is too much self-evident from the data that, like the author describes, Chinese patients might have different perspectives about the attributes compared to western patients. There needs some description of where this perspective came from.

Thank you. Your point is very helpful. A recent study found that the general spatial accessibility to hierarchical healthcare facilities in Shenzhen is unevenly distributed and concentrated (2). Also, the supply of healthcare resources at primary facilities is far from sufficient. This might reveal the reason why patients in China in our study were more concerned about blood monitoring. More frequent blood test monitoring is inconvenient for many patients who do not reside near their primary health care facility. Another study focused on oral anticoagulation adherence of patients in China suggested that consecutive INR monitoring was a major concern of patients, especially for those who are older, women, and have previous stroke history, as they are at a higher risk of bleeding and need more intensive INR monitoring(3). We have revised this part (Lines 399-407 of tracked version).

This is not understood. Failed to find a linkage between "holistic manner" and "even weight".: Chinese patients might consider all aspects of oral anticoagulant therapy in a more holistic manner and thus weight attributes for oral anticoagulants more evenly.

Thank you for the comment. As stated in the previous response, we would like to discuss it in the aspect of accessibility to healthcare facility instead of focusing on “Chinese patients might consider all aspects of oral anticoagulant more evenly”. We admit that the evidence that we provided for previous argument is not strong enough and have deleted it (Lines 397-399 of tracked version).

"The insignificance of out-of-pocket costs suggests that this population might be less concerned about the cost of treatment" might be better to be replaced by "The insignificance of out-of-pocket costs suggests that the current incremental cost is within the acceptable range of affordability". Preference would be influenced by changing out of pocket from the DCE, which was not tested.

Thank you for the suggestion. We have revised as suggested by the reviewer (Lines 427-428 of tracked version).


Reference

1.	Rowen D, Stevens K, Labeit A, Elliott J, Mulhern B, Carlton J, et al. Using a discrete-choice experiment involving cost to value a classification system measuring the quality-of-life impact of self-management for diabetes. Value Health. 2018;21(1):69-77.

2.	Tao Z, Cheng Y, Liu J. Hierarchical two-step floating catchment area (2SFCA) method: measuring the spatial accessibility to hierarchical healthcare facilities in Shenzhen, China. Int J Equity Health. 2020;19(1):164.

3.	Xiang X, Cao Y, Sun K, Song J, Tian Y, Yin Q, et al. Real world adherence to oral anticoagulant in non-valvular atrial fibrillation patients in China. Curr Med Res Opin. 2018;34(2):255-61.

Reviewer #4: This interesting study applied discrete-choice-experiment methods to investigate preferences and willingness to pay of Chinese patients with atrial fibrillation for oral anticoagulation therapy. Results indicate that patients were mostly concerned about the risks of acute myocardial infarction, stroke or systemic embolism and major bleeding, but were willing to pay a better drug with less risks if they could easily afford it.

Major comments:

- Methods:
* The risks of major bleeding, stroke/SE and myocardial infarction used as levels of the attributes, were based on 6 studies (references 23-28). However, were these studies based on outcome data in Chinese/Asian populations or not, given that risk estimates of other populations may be less generalizable to a Chinese population? Please discuss in the limitations.

Thank you for the comment. We have added this discussion in the limitations (Lines 446-451 of tracked version).

* How many patients were invited in order to include 506 patients in total?

Thank you. In order to reduce the work load for investigators, we did not record the actual total number of patients invited to participate. According to week reports of our investigators, from about 2-4 invited patients, there would be one patient who agreed to participate in the survey.

* Patients were randomly divided into two blocks with 8 scenarios for each. How was the randomisation process performed exactly?

Thank you for the comment. We used Ngene to randomly divide 16 generated scenarios into 2 blocks. Then, we flipped coins for patient allocation randomization. Patients who completed the consent form were allocated to different blocks according to the results of coin flip. Number indicated block A, emblem indicated block B.

* 37 patients did not choose the dominated one in the testing scenario and were considered as irrational. They were corrected by investigators before answering the remaining questions. What does this mean? Were they educated on the meaning of the attributes? Please elaborate.

Thank you for the question. A testing scenario (supplementary 1) was designed to test whether patients understand DCE questionnaire. In this scenario, drug A is obviously worse than drug B, because drug A has higher risks of suffering from side effect, including bleeding, MI, has higher risk of suffering from stroke, has food-drug interaction but no antidote, and has a higher price. If patients did not choose drug B, it indicated they might not understand this questionnaire. Investigators would immediately explain the meaning of levels of attributes to them. These patients were allowed to answer the questionnaire only when their understandings were confirmed by investigators. After investigators’ explanation, these patients were considered to have similar understanding as other patients did and were included in analyses. To assess whether the responses from these patients would influence our results, they were removed in one of the sensitivity analysis (supplementary 5). All the results were consistent with the main one. We revised sentences with “irrational” to make it clearer, and added number of these patients from both Beijing and Shenzhen (Lines 246-248, and table 2 of traced version).

- Results:
* In the methods, it is mentioned that most patients would have medical insurance, which is important as the authors mention that the out-of-pocket cost of NOACs for a patient with medical insurance is approximately 150-300 RMB (23-47 USD). Based on table 1, 28 patients were included without medical insurance. Did this patient subgroup influence the results?

Thank you for the question. We added one sensitivity analysis with all these 28 patients being excluded (supplementary 9). The results are consistent with main analysis.

* Scenarios with opt-out selected were removed from the main analysis. However, what were the driving attributes for these patients to not select an anticoagulant option?

Thank you for the question. Patients will choose opt-out only if they do not want to choose drug A nor drug B. Patients will weigh the advantages and disadvantages of two drugs according to the given combinations of attributes levels. Patients may be influenced by some crucial factors. For example, assume we included the risk of death as an attribute in our questionnaire, and patients really concerned about this attribute, if the risk of death for both drug A and drug B were at a very high level in one scenario, patients probably would not choose drug A nor drug B, no matter how different of the other attributes were. In this case, the risk of death is the driving attribute for patients to not choose an anticoagulant option. Consequently, the absolute value of coefficient of this attribute would also be much greater than others’. In our study, the difference between absolute values of all attributes’ coefficients were not too large. Patients might not be able to choose the best treatment based on the given combinations of levels of attributes. Therefore, it is hard to identify one or two actual attributes which drive the selection of opt-out option.

- Author summary:
* The current author summary is too large and the first two segments are almost identical to the abstract. I would recommend to summarize this section more and paraphrase duplicate statements.

Thank you for the suggestion. We have revised (Lines 76-121 of tracked version).

Minor comments:
* In the first paragraph of the introduction, the term 'drug/food interactions' is used, whereas in the rest of the manuscript, the term 'food-drug interactions' is used. I would recommend to use the same term throughout the manuscript.

Thank you for the comment. We have revised (Line 148 of tracked version).

- Grammatical/typing errors:
* Methods: "McFadden's Pseudo-R2" instead of "McFadden's Psuedo-R2"

Thank you for the comment. We have revised (Line 280 of tracked version).

* Discussion: "In addition, as apixaban and edoxaban have not yet been approved for use in China…" instead of "In addition, as apixaban andedoxaban have not yet been approved for use in China…"

Thank you for the comments. We have revised (Line 381 of tracked version).


Any attachments provided with reviews can be seen via the following link:
********

__________________________________________________
In compliance with data protection regulations, you may request that we remove your personal registration details at any time. (Use the following URL: https://www.editorialmanager.com/pmedicine/login.asp?a=r). Please contact the publication office if you have any questions.
